# The Skeleton and Biomineralization Mechanism as Part of the Innate Immune System of Stony Corals

**DOI:** 10.3389/fimmu.2022.850338

**Published:** 2022-02-25

**Authors:** Shani Levy, Tali Mass

**Affiliations:** ^1^ Department of Marine Biology, Leon H. Charney School of Marine Sciences, University of Haifa, Haifa, Israel; ^2^ Morris Kahn Marine Research Station, The Leon H. Charney School of Marine Sciences, University of Haifa, Sdot Yam, Israel

**Keywords:** stony corals, coral immune system, biomineralization, coral skeleton, immune genes, calicoblasts, calcification

## Abstract

Stony corals are among the most important calcifiers in the marine ecosystem as they form the coral reefs. Coral reefs have huge ecological importance as they constitute the most diverse marine ecosystem, providing a home to roughly a quarter of all marine species. In recent years, many studies have shed light on the mechanisms underlying the biomineralization processes in corals, as characterizing the calicoblast cell layer and genes involved in the formation of the calcium carbonate skeleton. In addition, considerable advancements have been made in the research field of coral immunity as characterizing genes involved in the immune response to pathogens and stressors, and the revealing of specialized immune cells, including their gene expression profile and phagocytosis capabilities. Yet, these two fields of corals research have never been integrated. Here, we discuss how the coral skeleton plays a role as the first line of defense. We integrate the knowledge from both fields and highlight genes and proteins that are related to biomineralization and might be involved in the innate immune response and help the coral deal with pathogens that penetrate its skeleton. In many organisms, the immune system has been tied to calcification. In humans, immune factors enhance ectopic calcification which causes severe diseases. Further investigation of coral immune genes which are involved in skeleton defense as well as in biomineralization might shed light on our understanding of the correlation and the interaction of both processes as well as reveal novel comprehension of how immune factors enhance calcification.

## Introduction

**Figure 1:**
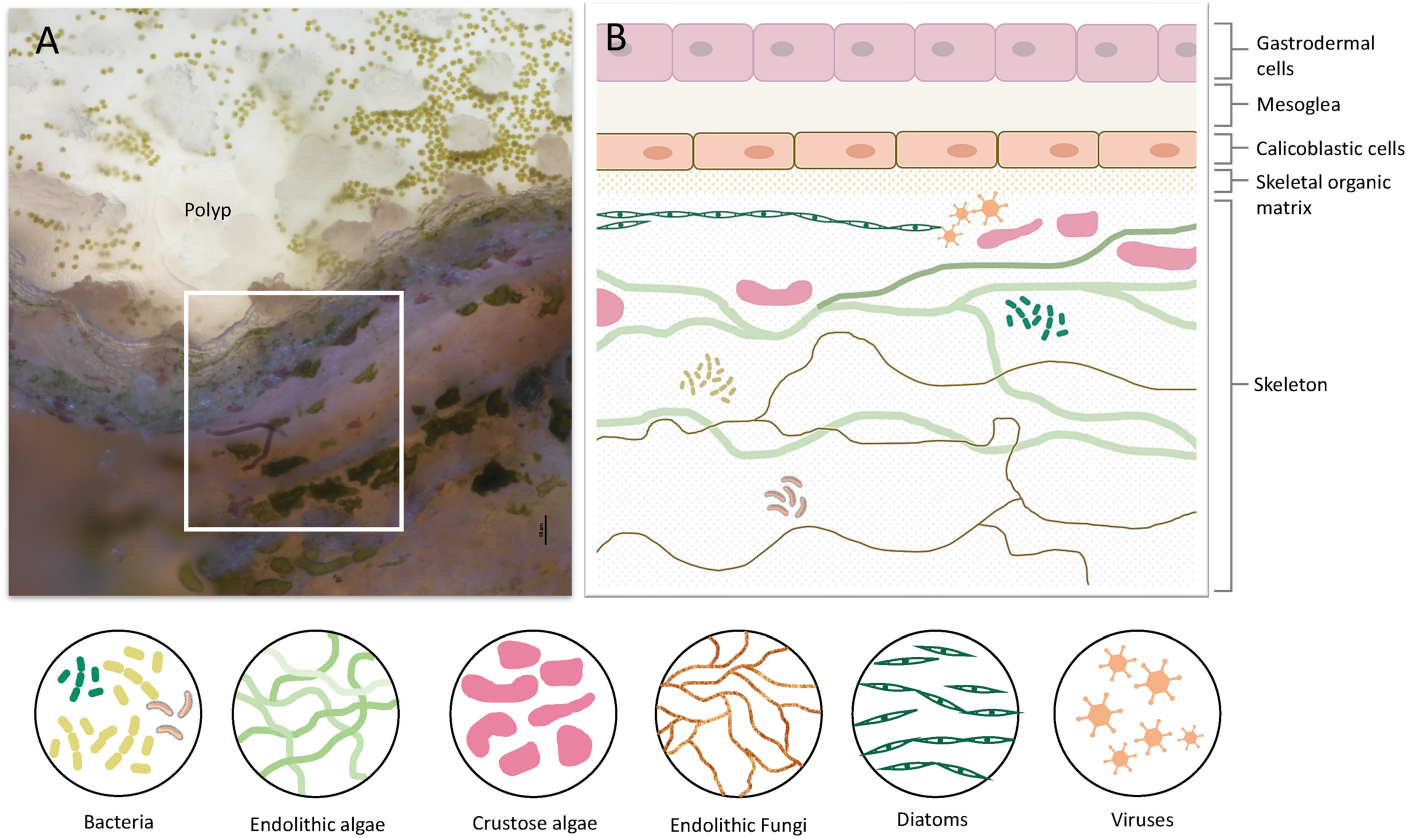
Coral tissue and skeleton layers and the diversity of organisms residing within the skeleton. **(A)** A cross-section of live *S. pistillata* coral. The polyp and coral tissue are on top (transparent white). Green dots in the tissue are the symbiotic algae (*Symbiodinium*). On the bottom half, there are skeleton layers with a diversity of microorganisms. **(B)** Illustration of the coral cell layers, and coral skeleton layers denoted in the white box in A, including the eukaryotic and prokaryotic microorganisms within the skeleton. The cell layer aligned with the skeletal organic matrix is the calicoblastic cell layer, which is involved in skeleton formation and in extracting molecules and proteins into the skeletal organic matrix.

Stony corals are among the most important calcifiers in the marine ecosystem. They hold significant ecological importance as they are the main builders of one of the most diverse and productive ecosystems in the ocean, the coral reefs (1, 2). Corals belong to the eumetazoan ancestor phylum Cnidaria, which are among the earliest metazoans to have evolved (3). Hence, they are significant in understanding the evolutionary origin as the early evolution of innate immunity (4, 5). Even though cnidarians lack some of the components of the adaptive immune system that are found in vertebrates, the sequencing of the first cnidarian genomes revealed a surprising immune complexity and a striking resemblance to bilaterian immune genes, with many ancestral immune components that have been lost in other invertebrates, such as *C. elegans*, and *D. melanogaster* (5, 6). Coral genome sequencing and comparative genomics have highlighted the immune gene repertoires of corals and underlined the evolution of specific immune genes in corals, such as an increased number of Toll-interleukin (TIR) proteins, and diversification of immune genes in different coral species, thus suggesting diverse adaptive roles for innate immune pathways in each species (7–9). Moreover, many studies showed up-regulation of immune genes following exposure to different stressors or pathogens (10–13), while others linked the immunity response to coral-algae symbiosis and showed the involvement of immune genes in the initiation of this symbiosis as well as in coral bleaching (14–17). Another rapidly evolving field is the coral microbiome which correlates coral health, resilience, and immune response to the holobiont and its microbiome (18–22). Even though the innate immune response of corals was extensively studied, the existence of immune cells in corals was an enigma. Although granular amoebocytes were observed in a few corals around wounds and lesions (16, 23), the genetic identification of specialized immune cells has only recently been described in the single-cell atlas of the coral *Stylophora pistillata* (24). The study revealed two distinct cell types with molecular signatures indicative of immune function. These cells express immune transcription factors such as NAFT and IRFs, and many genes involved in the innate immunity response such as the interleukin receptor, LSP binding proteins, Perforin, endonucleases, prosaponins, antimicrobial ApeC proteins, tyrosinase, and genes involved in the inflammatory response (24). Following these findings, Snyder et al. (25) identified and characterized phagocyte cells of the coral *Pocillopora damicornis* and the sea anemone *Nematostella vectensis*, and showed that the phagocytic cells engulf bacteria, fungal antigens, and beads. In addition to the immune cells, the innate immunity of corals involves other aspects of the defensive mechanism such as the cnidocytes (i.e., stinging cells), venom-producing gland cells (26–28), mucus secretion, and mucus-associated bacteria involved in the antimicrobial activity on the coral surface (21, 29–31). Another less studied aspect related to the stony coral’s immune system is their calcium carbonate exoskeleton that functions as an additional barrier to the external marine environment and hence might play a crucial role in the innate immune response. Stony coral polyps face the water column while the aboral epithelium, referred to as the calicoblastic layer, constantly produces the aragonite skeleton (**Figure 1**) (32). Corals grow continuously, by budding new polyps, and their aragonite skeleton expands accordingly. In addition, new layers of aragonite are continuously deposited and accumulate on top of the old layers. Although the exact mechanism of the biomineralization process remains elusive, our understanding of the molecular mechanism underlying this process has greatly advanced (33–37). Proteomic analysis of the skeletal organic matrix from three different coral species revealed an assemblage of adhesion and structural proteins, transmembrane proteins, proteins containing known extracellular matrix (ECM) domains, as well as highly acidic proteins that were suggested to play a role in calcium carbonate nucleation (38–41). Furthermore, the first stony coral single-cell atlas characterized the gene expression profile of the cells involved in the formation of the coral skeleton (calicoblasts) and revealed more than 700 genes that are specifically expressed in the calicoblasts of the juvenile primary polyp and the adult coral.

Although research of both biomineralization and immunity in corals have advanced considerably over recent years, as of yet, these two fields have not been integrated. Here, we underline the immunological basis of corals in a biomineralization context by reviewing and integrating the knowledge in both fields as well as highlighting immune genes expressed in the cells that form the coral skeleton or found in the skeleton itself.

## The Coral Skeleton as the First Barrier Against Pathogens

**Figure 2:**
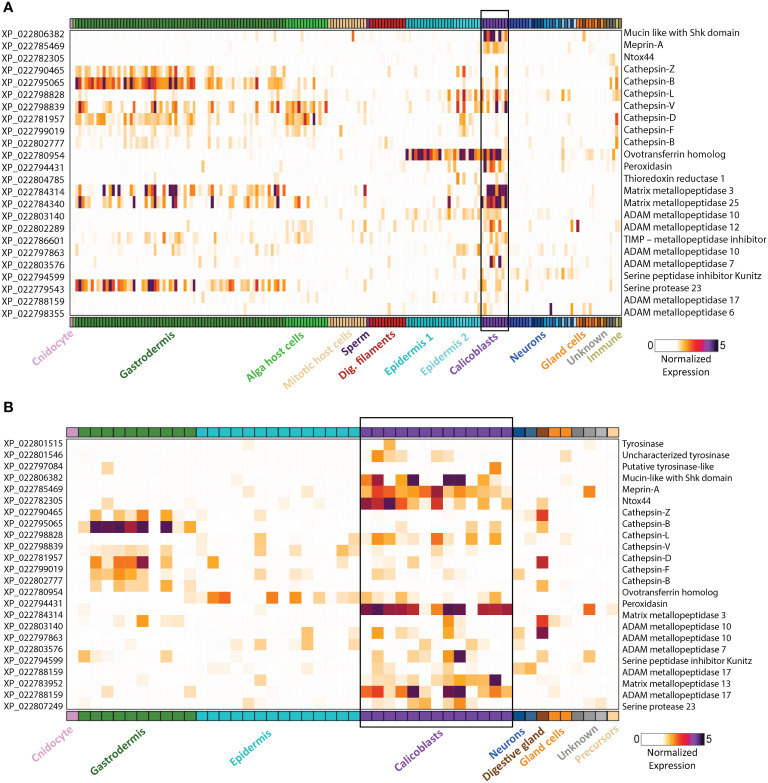
Gene expression heatmap for selected genes that might play a dual role in biomineralization and immunity. Expression levels (fold-change) are shown across all cell types of the stony coral *S. pistillata*. Calicoblast cells are in purple and are emphasized with a black box **(A)** Adult gene expression heatmap of selected genes. **(B)** Primary polyp gene expression heatmap of selected genes. Heatmaps were created using the interactive database https://sebe-lab.shinyapps.io/Stylophora_cell_atlas/ (24).

The coral life cycle involves a planktonic larva and a benthic adult. These two phases are separated by settlement and metamorphosis, two critical stages in coral development, during which some of the epidermal cells are transformed into calicoblast cells that immediately start with rapid skeleton deposition (32). This rapid process is important for coral adherence to the substrate, as well as in creating a protective environment, in the form of an aragonite skeleton, for the soft and vulnerable polyp (42). This process might be involved in the production and secretion of anti-microbial factors to clear the surface and prevent possible pathogenic infections. As in many other marine organisms, the exoskeleton is a physical barrier that protects the animal from the outside world and serves as the first line of defense. When coral polyps sense a physical threat (e.g., predators, strong currents, suspended sediment), they contract into their aragonite calyx in order to avoid the danger (43). In addition to physical protection, exoskeletons constitute biochemical protection, and in many organisms the exoskeleton is rich in antimicrobial molecules, enzymes, and toxins (44–47). Coral exoskeletons sustain diverse eukaryotic and prokaryotic microorganisms such as fungi, endolithic algae, viruses, and bacteria (**Figure 1**) (48–51). While these organisms are part of the holobiont and can produce metabolites and antimicrobial compounds that help the coral control its skeleton microbiota, others can be pathogenic and might use the skeleton to invade and penetrate the coral tissue (51, 52). Thus, the exoskeleton and the calicoblastic layer aligned to it (**Figure 1B**) might have an additional immune protection role against the invaders. One element of immune protection found in exoskeletons is melanin (53, 54). As a polymer, melanin can strengthen tissue and exoskeletons and improve their ability to act as physical barriers against the penetration of parasites (55). Furthermore, melanin can introduce potent antimicrobial activity by inhibiting lytic enzymes produced by microorganisms (56). In Arthropods, melanin deposits in the exocuticle play an important role in increasing the immune protection of the exoskeleton (53). In stony coral, melanin has been detected in granular cells and specifically in the tissue around wounds, during the healing of wounds in the coral *Porites cylindrica* (16). In the common sea fan *Gorgonia ventalina*, melanin has been observed around tissue lesions formed by the invasion of the pathogenic fungi *Aspergillus sydowii* (23). The fungal hyphae have been observed in the coral’s axial skeleton, with a thick melanin layer formed around the infection that serves as a barrier preventing the *A. sydowii* hyphae from contacting its tissue. In addition, an increase in pigmented calcium carbonate sclerites (the skeletal elements of soft corals) was observed, which gives the lesions their distinct, dark coloration (23). Proteins involved in melanin production such as tyrosinase have been detected in molluscan mantle transcriptomes and shells and were suggested to be involved in exoskeleton fabrication and hardening (57–59). In *S. pistillata*, tyrosinase genes were found to be expressed in the adult immune cells, while three different tyrosinase genes were expressed in the juvenile primary polyp calicoblastic cells (**Figure 2B** and **Table S1**) (24). One of these genes is a tyrosinase-like (XP_022797084.1) that possesses four ShK toxin domains, which are potassium channel blockers that were first isolated from the sea anemone *Stichodactyla helianthus* venom (60). While most genes with ShK domains showed high expression in *S. pistillata* cnidocytes (stinging cells) and gland cells, two extracellular genes containing ShK domains showed high specific expression in both adult and primary polyp calicoblasts (**Figure 2**) (24). One is a homolog of the protein meprin A (XP_022785469.1), a metallopeptidase with the ability to cleave various substrates, degrade ECM proteins, process proinflammatory cytokines, and promote leukocyte infiltration (61). Therefore, it might have similar functions in remodeling the corals’ skeletal organic matrix and initiating the inflammatory response once pathogens are detected. The second gene is a mucin-like protein with two Shk domains (XP_022806382.1), which could be secreted into the skeletal organic matrix and serve as a toxin.

Another toxic candidate gene, highly expressed in the primary polyp calicoblasts, is Ntox44 (XP_022782305.1) (**Figure 2B**) (24). This gene is a homolog of a bacterial secreted RNase toxin with potential antimicrobial function. Hence, it might play a role in clearing the substrate while the larva metamorphoses into a primary polyp and starts calcifying its initial skeleton. Ramos-Silva et al. (38) reported on an additional toxin-like protein, (B7W114), found in the *Acropora millepora* skeletal proteome, which corresponds to a secreted protein with high similarity to the SE-cephalotoxin from the cephalopod *Sepia esculenta.*

In addition to the secretion of toxins and antimicrobial compounds into the skeleton, the biomineralization process can act as a direct immune defense mechanism. The most familiar example is the pearl formation in mollusks, in which the animal uses its calcification ability against irritant foreign bodies, parasites, or other pathogens by creating a calcium carbonate structure (62). As for corals, there is only one document (as per our knowledge) of a calcification defense mechanism shown in response to a fungal invasion (63). Le Campion et al. demonstrated that the stony coral *Porites lobata* responds to fungi penetrating its skeleton by the deposition of calcium carbonate, to form skeleton thickness that will prevent the fungi from reaching the polyp tissue (63). Further investigation of this interesting phenomenon in corals might reveal the correlation between the self and non-self-recognition, the innate immune response, and the calcification processes. Additionally, further molecular investigation of this phenomenon could shed light on genes which are involved in both processes, the innate immune response and calcification.

## Genes With Potential Dual Function in the Coral’s Innate Immune Response and in Biomineralization

In recent years, increasing evidence regarding the integration of biomineralization and immunity has come to light, including proteins with dual function in both (64–66). In humans and other mammalians, immunity and calcification have been tightly connected, as many studies have shown that immune cells are closely associated with ectopic calcification as in the development of atherosclerosis, vascular calcification, chronic kidney disease, breast cancer, etc (67–69). It has been demonstrated that vascular calcification is part of the immune response and involves many factors and genes of the innate and adaptive immune system (69–71). The lack of effective therapy for ectopic calcification is an indicator of the complexity of its mechanism as well as the significance of understanding the interaction between the immune response and calcification (69, 72). Uncovering the individual contribution of immune genes to enhanced calcification, would improve our understanding of the inflammation dependent mechanisms of ectopic calcification, and could offer new diagnosis tools as well as therapeutic treatments for the involved diseases.

To the best of our knowledge, there are no studies that link between the immune response and calcification in corals. To create a database of genes with potential immune and biomineralization functions, we explored the genes that are expressed in the calicoblastic cells, (24), the proteins found in the skeletal organic matrix proteomes of several corals (38, 40, 73) and searched the available literature for known functions of their homologs in other organisms. This data can serve as a database for further investigations of the molecular mechanisms that underlie the response of corals to pathogens that penetrate their skeleton. The whole gene list is available in **Table S1**.

We found that genes that are known to be involved in vascular calcification, such as CD36, DOCK1, DSPP, and Perforin (74–76), are expressed in the coral immune cells but not in the calicoblasts. This might imply that these genes do not play a role in coral calcification. However, immune cells that express these genes might enhance calcification in corals in a similar manner to the enhancement of vascular calcification by macrophages (69, 71). It will be interesting to investigate this issue in corals, for example, during the wound healing processes, in which immune cells might migrate toward the wound (16), help repair the tissue and protect it from pathogens and additionally enhance skeleton precipitation to repair the damaged skeleton.

In addition, we found that many cathepsin genes are expressed in both calicoblasts and in the immune Cells (**Figure 2** and **Table S1**). Cathepsins are multifunctional enzymes involved in many biological processes such as lysosomal protein recycling, digestion, wound healing, bone remodeling, reproduction, and innate immune response (77). Cathepsin L, which is expressed in both immune and calicoblast cells, is known to be a multifunctional protein involved in the immune response of fish and mollusks (78–80), in biomineralization (81), and in bone and cartilage resorption in humans (82); Cathepsin D, which is expressed only in the immune cells of *S. pistillata*, is a membrane-associated acidic protease, familiar with macrophage endosomes (83), also involved in cardiovascular calcification (84); and Cathepsin V, which is expressed only in the calicoblasts, is known to promote vascular calcification in humans (85).

Another interesting protein is the ovotransferrin that was found to have a dual role in avian eggshell formation (86). It was first identified as an antibacterial and antifungal protein (87, 88) and later was found to have a role in the biomineralization processes as it was expressed in the initial stage of shell biomineralization and was localized to the sites of calcite nucleation (44). Furthermore, the addition of the purified protein *in-vitro* results in a large modification of the calcium carbonate crystals morphology. In corals, we found a homolog ovotransferrin gene (XP_022780954.1) with high expression in both adult and primary polyp calicoblasts (**Figure 2** and **Table S1**) (24). Therefore, we suggest that this gene potentially can be involved in coral biomineralization and serves as a bacteriostatic filter.

Another protein that might have a dual function is Peroxidasin (XP_022794431.1). In the human myofibroblasts, peroxidasin is secreted into the extracellular space where it becomes organized into a fibril-like network and colocalizes with fibronectin to form the ECM (89). It catalyzes sulfilimine bond formation in collagen IV and catalyzes hydrogen peroxide (H_2_O_2_) into hypochlorous acid (HOCl). An excessive peroxidasin activity, allows free oxidizing hypohalous acid to accumulate and produce intended or unintended toxicity (90). The high reactivity of the hypochlorous acid toward a variety of biological molecules, cause oxidative damage to pathogens proteins and contribute to the killing of pathogens as was demonstrated in neutrophils (91). Since in *S. pistillata* peroxidasin is specifically expressed in the adult and primary polyp calicoblasts (**Figure 2**) (24), we suggest that it is secreted into the skeletal organic matrix where it might generate fibril-like network and in addition, produce hypohalous acids with toxic activity.

Next, we explored all proteins found in scleractinian skeleton proteomes (38, 40, 73) and looked for proteins with a possible immune function. One such protein is the sacsin protein (40), that acts as a regulator of the Hsp70 chaperone machinery (92, 93). While the mammalian sacsin was studied in association with a neural disorder (92), the sacsin homolog in fish was reported to be involved in the innate antiviral immune response in several fish species (94–96).

A second protein is thioredoxin reductase 1, cytoplasmic (XP_022804785.1), its human homolog mediates cell death induced by a combination of interferon-beta and retinoic acid (97). It also induces actin and tubulin polymerization, leading to the formation of cell membrane protrusions (98). Cell membrane protrusions were observed in the coral calicoblastic cell layer and are thought to be essential structures for coral skeleton formation (99, 100). In addition, this protein might be involved in the induction of calicoblasts apoptosis, in case of infection.

Furthermore, a few proteases were found as well (38, 40, 73). These proteases are thought to be involved in digestion and modeling the skeletal organic matrix as a scaffold for the calcium carbonate skeleton and in processing and activating other bioactive molecules and proteins involved in the biomineralization process (101, 102). Proteases and specifically serine proteases are also known to be key mediators of the innate immune response as they act as processing enzymes of pro-inflammatory cytokines and other enzymes related to the inflammatory response (103–105). Additionally, many proteases, including matrix metallopeptidases and protease inhibitors are expressed in *S. pistillata* calicoblasts as well (**Figure 2** and **Table S1**) (24). Metalloproteases, such as MMP-25, regulate the innate immune response through the NF-kB signaling in mice (106). A homolog of MMP-25 is highly expressed in the adult calicoblasts of *S. pistillata* (**Figure 2A**) (24). Since metallopeptidases hydrolyze and process a large number of substrates, they might be involved in remodeling the skeletal organic matrix scaffold of the coral skeleton or in the interaction of the calicoblastic cell layer with the skeleton ECM. In-addition, some of the metalloproteases might be involved in processing and activating factors involved in biomineralization such as the acidic proteins involved in nucleation or factors involved in the innate immune response such as pro-inflammatory cytokines and chemokines, growth factors and other receptors’ ligands.

## Discussion

The coral skeleton serves as a firm structure for animal protection, and as the first line of defense against invaders and pathogens. In order to protect the animal from these parasites and pathogens, the exoskeleton must include antimicrobial molecules and toxins. Some are produced by the symbionts inside the skeleton and help the coral control its skeleton biota. Others, most likely, are extracted by the coral itself, through the tissue that forms the skeleton, the calicoblastic layer. In this review, we highlighted genes and proteins that might serve as toxins or bacteriostatic molecules as well as genes and proteins that are known to play a role in the immune response and are found either in the calicoblastic cells or in the skeleton itself (**Figure 2** and **Table S1**). Further exploration of the role of these genes along the process of biomineralization can illuminate how corals deal with pathogens that penetrate their skeleton as well as reveal immune genes that might be involved in the biomineralization process or enhance calcification.

Stony corals belong to the Anthozoa class in the Cnidaria phylum, a sister group of Bilateria. As stony corals are the only cnidarians that build an exoskeleton, they hold an interesting and important key position in our understanding of the evolution of the immune system and its involvement in calcification. Understanding the mechanisms that correlate immunity and calcification, and revealing the role of genes shared by both, is a valid point that may help shed light on these complex mechanisms. It can reveal novel etiologies of ectopic calcification involved in severe diseases and chronic disorders such as vascular calcification, atherosclerosis, osteoarthritis, kidney stones and several cancers. Thus, it can provide new tools for diagnosis and treatments for these common pathologies.

As a whole, we tried to review and integrate the data obtained in two important and enhanced fields in coral research and create a valuable database for further research to better understand how biomineralization and the innate immune system are involved, and which factors are shared by both.

## Author Contributions

SL and TM contributed to the conception and design of the article and interpreting the relevant literature. SL designed the figures and wrote the first draft, with inputs from TM. All authors contributed to the article and approved the submitted version.

## Funding

This work has received funding from the European Research Council (ERC) under the European Union’s Horizon 2020 research and innovation programme (grant agreement No 755876).

## Conflict of Interest

The authors declare that the research was conducted in the absence of any commercial or financial relationships that could be construed as a potential conflict of interest.

## Publisher’s Note

All claims expressed in this article are solely those of the authors and do not necessarily represent those of their affiliated organizations, or those of the publisher, the editors and the reviewers. Any product that may be evaluated in this article, or claim that may be made by its manufacturer, is not guaranteed or endorsed by the publisher.

## References

[1] Moberg F Folke C. Ecological Goods and Services of Coral Reef Ecosystems. Ecol Economics (1999) 29(2):215–33. doi: 10.1016/S0921-8009(99)00009-9

[2] Hughes TP Barnes ML Bellwood DR Cinner JE Cumming GS Jackson JBC. Coral Reefs in the Anthropocene. Nature (2017) 546(7656):82–90. doi: 10.1038/nature22901 28569801

[3] Berntson EA France SC Mullineaux LS. Phylogenetic Relationships within the Class Anthozoa (Phylum Cnidaria) Based on Nuclear 18S rDNA Sequences. Mol Phylogenet Evol (1999) 13(2):417–33. 10.1006/mpev.1999.0649 10603268

[4] Genikhovich G Technau U. The Starlet Sea Anemone Nematostella Vectensis: An Anthozoan Model Organism for Studies in Comparative Genomics and Functional Evolutionary Developmental Biology. Cold Spring Harbor Protoc (2009) 2009(9):pdb. emo129. doi: 10.1101/pdb.emo129 20147257

[5] Augustin R Bosch TCG. Cnidarian Immunity: A Tale of Two Barriers. New York: Springer US (2010) p. 1–16. 10.1007/978-1-4419-8059-5_1 21528690

[6] Miller DJ Hemmrich G Ball EE Hayward DC Khalturin K Funayama N. The Innate Immune Repertoire in Cnidaria - Ancestral Complexity and Stochastic Gene Loss. Genome Biol (2007) 8(4):R59. doi: 10.1186/gb-2007-8-4-r59 17437634 PMC1896004

[7] Shinzato C Shoguchi E Kawashima T Hamada M Hisata K Tanaka M. Using the Acropora Digitifera Genome to Understand Coral Responses to Environmental Change. Nature (2011) 476(7360):320–3. doi: 10.1038/nature10249 21785439

[8] Voolstra CR Li Y Liew YJ Baumgarten S Zoccola D Flot J-F. Comparative Analysis of the Genomes of Stylophora Pistillata and Acropora Digitifera Provides Evidence for Extensive Differences Between Species of Corals. Sci Rep (2017) 7(1). doi: 10.1038/s41598-017-17484-x PMC5730576 29242500

[9] Cunning R Bay RA Gillette P Baker AC Traylor-Knowles N. Comparative Analysis of the Pocillopora Damicornis Genome Highlights Role of Immune System in Coral Evolution. Sci Rep (2018) 8(1). doi: 10.1038/s41598-018-34459-8 PMC6208414 30382153

[10] Vidal-Dupiol J Ladrière O Destoumieux-Garzón D Sautière P-E Meistertzheim A-L Tambutté E. Innate Immune Responses of a Scleractinian Coral to Vibriosis. J Biol Chem (2011) 286(25):22688–98. doi: 10.1074/jbc.M110.216358 PMC3121412 21536670

[11] Libro S Kaluziak ST Vollmer SV. RNA-Seq Profiles of Immune Related Genes in the Staghorn Coral Acropora Cervicornis Infected With White Band Disease. PloS One (2013) 8(11):e81821. doi: 10.1371/journal.pone.0081821 24278460 PMC3836749

[12] Van De Water JAJM Ainsworth TD Leggat W Bourne DG Willis BL Van Oppen MJH. The Coral Immune Response Facilitates Protection Against Microbes During Tissue Regeneration. Mol Ecol (2015) 24(13):3390–404. doi: 10.1111/mec.13257 26095670

[13] Anderson DA Walz ME Weil E Tonellato P Smith MC. RNA-Seq of the Caribbean Reef-Building Coralorbicella Faveolata(Scleractinia-Merulinidae) Under Bleaching and Disease Stress Expands Models of Coral Innate Immunity. PeerJ (2016) 4:e1616. doi: 10.7717/peerj.1616 26925311 PMC4768675

[14] Kvennefors ECE Leggat W Kerr CC Ainsworth TD Hoegh-Guldberg O Barnes AC. Analysis of Evolutionarily Conserved Innate Immune Components in Coral Links Immunity and Symbiosis. Dev Comp Immunol (2010) 34(11):1219–29. doi: 10.1016/j.dci.2010.06.016 20600272

[15] Palmer CV Bythell JC Willis BL. Levels of Immunity Parameters Underpin Bleaching and Disease Susceptibility of Reef Corals. FASEB J (2010) 24(6):1935–46. doi: 10.1096/fj.09-152447 20124432

[16] Palmer CV McGinty ES Cummings DJ Smith SM Bartels E Mydlarz LD. Corals Use Similar Immune Cells and Wound-Healing Processes as Those of Higher Organisms. PloS One (2011) 6(8):e23992 21887359 10.1371/journal.pone.0023992 PMC3161096

[17] Mohamed AR Cumbo V Harii S Shinzato C Chan CX Ragan MA. The Transcriptomic Response of the Coralacropora Digitiferato a Competentsymbiodiniumstrain: The Symbiosome as an Arrested Early Phagosome. Mol Ecol (2016) 25(13):3127–41. doi: 10.1111/mec.13659 27094992

[18] Bourne DG Morrow KM Webster NS. Insights Into the Coral Microbiome: Underpinning the Health and Resilience of Reef Ecosystems. Annu Rev Microbiol (2016) 70(1):317–40. doi: 10.1146/annurev-micro-102215-095440 27482741

[19] Van Oppen MJH Blackall LL. Coral Microbiome Dynamics, Functions and Design in a Changing World. Nat Rev Microbiol (2019) 17(9):557–67. doi: 10.1038/s41579-019-0223-4 31263246

[20] Connelly MT McRae CJ Liu P-J Traylor-Knowles N. Lipopolysaccharide Treatment Stimulates Pocillopora Coral Genotype-Specific Immune Responses But Does Not Alter Coral-Associated Bacteria Communities. Dev Comp Immunol (2020) 109:103717. doi: 10.1016/j.dci.2020.103717 32348787

[21] Osman EO Suggett DJ Voolstra CR Pettay DT Clark DR Pogoreutz C. Coral Microbiome Composition Along the Northern Red Sea Suggests High Plasticity of Bacterial and Specificity of Endosymbiotic Dinoflagellate Communities. Microbiome (2020) 8(1):1–16. doi: 10.1186/s40168-019-0776-5 32008576 PMC6996193

[22] Santoro EP Borges RM Espinoza JL Freire M Messias CS Villela HD. Coral Microbiome Manipulation Elicits Metabolic and Genetic Restructuring to Mitigate Heat Stress and Evade Mortality. Sci Adv (2021) 7(33):eabg3088. doi: 10.1126/sciadv.abg3088 34389536 PMC8363143

[23] Mydlarz LD Holthouse SF Peters EC Harvell CD. Cellular Responses in Sea Fan Corals: Granular Amoebocytes React to Pathogen and Climate Stressors. PloS One (2008) 3(3):e1811. doi: 10.1371/journal.pone.0001811 18364996 PMC2267492

[24] Levy S Elek A Grau-Bové X Menéndez-Bravo S Iglesias M Tanay A. A Stony Coral Cell Atlas Illuminates the Molecular and Cellular Basis of Coral Symbiosis, Calcification, and Immunity. Cell (2021) 184(11):2973–87.e2918. doi: 10.1016/j.cell.2021.04.005 33945788 PMC8162421

[25] Snyder GA Eliachar S Connelly MT Talice S Hadad U Gershoni-Yahalom O. Functional Characterization of Hexacorallia Phagocytic Cells. Front Immunol (2021) 12. doi: 10.3389/fimmu.2021.662803 PMC8350327 34381444

[26] Tardent P. The Cnidarian Cnidocyte, a Hightech Cellular Weaponry. BioEssays (1995) 17(4):351–62. doi: 10.1002/bies.950170411

[27] Moran Y Genikhovich G Gordon D Wienkoop S Zenkert C Özbek S. Neurotoxin Localization to Ectodermal Gland Cells Uncovers an Alternative Mechanism of Venom Delivery in Sea Anemones. Proc R Soc B: Biol Sci (2012) 279(1732):1351–8. doi: 10.1098/rspb.2011.1731 PMC3282367 22048953

[28] Schmidt CA Daly NL Wilson DT. Coral Venom Toxins. Front Ecol Evol (2019) 7(320). doi: 10.3389/fevo.2019.00320

[29] Goreau T. Histochemistry of Mucopolysaccharide-Like Substances and Alkaline Phosphatase in Madreporaria. Nature (1956) 177(4518):1029–30. doi: 10.1038/1771029a0

[30] Richardson L. Coral Diseases: What is Really Known? Trends Ecol Evol (1998) 13(11):438–43. doi: 10.1016/S0169-5347(98)01460-8 21238385

[31] Shnit-Orland M Kushmaro A. Coral Mucus-Associated Bacteria: A Possible First Line of Defense. FEMS Microbiol Ecol (2009) 67(3):371–80. 10.1111/j.1574-6941.2008.00644.x 19161430

[32] Wainwright SA. Skeletal Organization in the Coral, Pocillopora Damicornis. J Cell Sci (1963) s3-104(66):169–83. doi: 10.1242/jcs.s3-104.66.169

[33] Bertucci A Tambutté S Supuran CT Allemand D Zoccola D. A New Coral Carbonic Anhydrase in Stylophora Pistillata. Marine Biotechnol (2011) 13(5):992–1002. doi: 10.1007/s10126-011-9363-x 21318259

[34] Mass T Drake J Haramaty L Dongun Kim J Zelzion E Bhattacharya D. Cloning and Characterization of Four Novel Coral Acid-Rich Proteins That Precipitate Carbonates In Vitro. Curr Biol (2013) 23(12):1126–31. doi: 10.1016/j.cub.2013.05.007 23746634

[35] Mass T Putnam HM Drake JL Zelzion E Gates RD Bhattacharya D. ). “Temporal and Spatial Expression Patterns of Biomineralization Proteins During Early Development in the Stony Coralpocillopora Damicornis. Proceedings of the Royal Society B (2016) 283:1829. doi: 10.1098/rspb.2016.0322 PMC4855386 27122561

[36] Barron ME Thies AB Espinoza JA Barott KL Hamdoun A Tresguerres M. A Vesicular Na+/Ca2+ Exchanger in Coral Calcifying Cells. PloS One (2018) 13(10):e0205367. doi: 10.1371/journal.pone.0205367 30379874 PMC6209159

[37] Zaquin T Malik A Drake JL Putnam HM Mass T. Evolution of Protein-Mediated Biomineralization in Scleractinian Corals. Front Genet (2021) 12(52). doi: 10.3389/fgene.2021.618517 PMC7902050 33633782

[38] Ramos-Silva P Kaandorp J Huisman L Marie B Zanella-Cléon I Guichard N. The Skeletal Proteome of the Coral Acropora Millepora: The Evolution of Calcification by Co-Option and Domain Shuffling. Mol Biol Evol (2013) 30(9):2099–112. doi: 10.1093/molbev/mst109 PMC3748352 23765379

[39] Takeuchi T Yamada L Shinzato C Sawada H Satoh N. Stepwise Evolution of Coral Biomineralization Revealed With Genome-Wide Proteomics and Transcriptomics. PloS One (2016) 11(6):e0156424. doi: 10.1371/journal.pone.0156424 27253604 PMC4890752

[40] Peled Y Drake JL Malik A Almuly R Lalzar M Morgenstern D. Optimization of Skeletal Protein Preparation for LC–MS/MS Sequencing Yields Additional Coral Skeletal Proteins in Stylophora Pistillata. BMC Materials (2020) 2(1). doi: 10.1186/s42833-020-00014-x PMC7115838 32724895

[41] Drake JL Varsano N Mass T. Genetic Basis of Stony Coral Biomineralization: History, Trends and Future Prospects. ” J Struct Biol (2021) 107782. doi: 10.1016/j.jsb.2021.107782 34455069 PMC7611647

[42] Mao X Nie Y Huang Y Ji H Li X. A Radial Distribution of Calices in Coral Skeleton of Pocillopora Verrucosa (Ellis and Solande ) Against Ocean Currents. Marine Biol (2021) 168(12):1–7. doi: 10.1007/s00227-021-03982-0

[43] Barnes R. Invertebrate Zoology. Fifth Edit. Fort Worth, TX: Harcourt Brace Jovanovich College Publishers (1987).

[44] Gautron J Hincke MT Panheleux M Garcia-Ruiz JM Boldicke T Nys Y. Ovotransferrin is a Matrix Protein of the Hen Eggshell Membranes and Basal Calcified Layer. Connective Tissue Res (2001) 42(4):255–67. doi: 10.3109/03008200109016840 11913770

[45] Mine Y Oberle C Kassaify Z. Eggshell Matrix Proteins as Defense Mechanism of Avian Eggs. J Agric Food Chem (2003) 51(1):249–53. doi: 10.1021/jf020597x 12502416

[46] Mansilla AY Albertengo L Rodríguez MS Debbaudt A Zúñiga A Casalongué CA. Evidence on Antimicrobial Properties and Mode of Action of a Chitosan Obtained From Crustacean Exoskeletons on Pseudomonas Syringae Pv. Tomato DC3000. Appl Microbiol Biotechnol (2013) 97(15):6957–66. doi: 10.1007/s00253-013-4993-8 23703326

[47] Jin C Liu X-J Li J-L. A Kunitz Proteinase Inhibitor (HcKuPI) Participated in Antimicrobial Process During Pearl Sac Formation and Induced the Overgrowth of Calcium Carbonate in Hyriopsis Cumingii. Fish Shellfish Immunol (2019) 89:437–47. doi: 10.1016/j.fsi.2019.04.021 30980916

[48] Shashar N Stambler N. Endolithic Algae Within Corals-Life in an Extreme Environment. J Exp Marine Biol Ecol (1992) 163(2):277–86. doi: 10.1016/0022-0981(92)90055-F

[49] Glynn PW Manzello DP. Bioerosion and Coral Reef Growth: A Dynamic Balance. In: Coral Reefs in the Anthropocene. Springer (2015). p. 67–97.

[50] Soler-Hurtado MM Sandoval-Sierra JV Machordom A Diéguez-Uribeondo J. Aspergillus Sydowii and Other Potential Fungal Pathogens in Gorgonian Octocorals of the Ecuadorian Pacific. PloS One (2016) 11(11):e0165992. doi: 10.1371/journal.pone.0165992 27902710 PMC5130190

[51] Pernice M Raina J-B Rädecker N Cárdenas A Pogoreutz C Voolstra CR. Down to the Bone: The Role of Overlooked Endolithic Microbiomes in Reef Coral Health. ISME J (2020) 14(2):325–34. doi: 10.1038/s41396-019-0548-z PMC6976677 31690886

[52] Ricci F Rossetto Marcelino V Blackall LL Kühl M Medina M Verbruggen H. Beneath the Surface: Community Assembly and Functions of the Coral Skeleton Microbiome. Microbiome (2019) 7(1). doi: 10.1186/s40168-019-0762-y PMC6909473 31831078

[53] Moret Y Moreau J. The Immune Role of the Arthropod Exoskeleton. Invertebrate Survival J (2012) 9(2):200–6.

[54] Shattock SG Dudgeon LS. (1908). Observations Upon Phagocytosis Carried Out by Means of Melanin to Ascertain More Particularly Whether the Opsonic Index is Identical With the Hæmophagocytic Index, in: Proceedings of the Royal Society of London. Series B, Containing Papers of a Biological Character. 80(538):165–81.

[55] St. Leger RJ Cooper RM Charnley AK. The Effect of Melanization of Manduca Sexta Cuticle on Growth and Infection by Metarhizium Anisopliae. J Invertebrate Pathol (1988) 52(3):459–70. doi: 10.1016/0022-2011(88)90059-6

[56] Mackintosh JA. The Antimicrobial Properties of Melanocytes, Melanosomes and Melanin and the Evolution of Black Skin. J Theor Biol (2001) 211(2):101–13. doi: 10.1006/jtbi.2001.2331 11419954

[57] Zhang C Xie L Huang J Chen L Zhang R. A Novel Putative Tyrosinase Involved in Periostracum Formation From the Pearl Oyster (Pinctada Fucata). Biochem Biophys Res Commun (2006) 342(2):632–9. doi: 10.1016/j.bbrc.2006.01.182 16488396

[58] Nagai K Yano M Morimoto K Miyamoto H. Tyrosinase Localization in Mollusc Shells. Comp Biochem Physiol Part B: Biochem Mol Biol (2007) 146(2):207–14. doi: 10.1016/j.cbpb.2006.10.105 17150393

[59] Aguilera F McDougall C Degnan BM. Evolution of the Tyrosinase Gene Family in Bivalve Molluscs: Independent Expansion of the Mantle Gene Repertoire. Acta biomaterialia (2014) 10(9):3855–65. doi: 10.1016/j.actbio.2014.03.031 24704693

[60] Castañeda O Sotolongo V Amor AM Stöcklin R Anderson AJ Harvey AL. Characterization of a Potassium Channel Toxin From the Caribbean Sea Anemone Stichodactyla Helianthus. Toxicon (1995) 33(5):603–13. doi: 10.1016/0041-0101(95)00013-C 7660365

[61] Kaushal GP Haun RS Herzog C Shah SV. Meprin A Metalloproteinase and its Role in Acute Kidney Injury. Am J Physiol-Renal Physiol (2013) 304(9):F1150–8. doi: 10.1152/ajprenal.00014.2013 PMC3651633 23427141

[62] Gliński Z Jarosz J. Molluscan Immune Defenses. Archivum immunologiae Therapiae Experimentalis (1997) 45(2-3):149–55. 9597080

[63] Le Campion-Alsumard T Golubic S Priess K. Fungi in Corals: Symbiosis or Disease? Interaction Between Polyps and Fungi Causes Pearl-Like Skeleton Biomineralization. Marine Ecol Prog Ser (1995), 137–47. doi: 10.3354/meps117137

[64] Réhault-Godbert S Hervé-Grépinet V Gautron J Cabau C Nys Y Hincke M. 9 - Molecules Involved in Chemical Defence of the Chicken Egg. In: Nys Y Bain M Van Immerseel F, editors. Improving the Safety and Quality of Eggs and Egg Products. Woodhead Publishing (2011). 80(538):165–81.

[65] Zhao X Wang Q Jiao Y Huang R Deng Y Wang H. Identification of Genes Potentially Related to Biomineralization and Immunity by Transcriptome Analysis of Pearl Sac in Pearl Oyster Pinctada Martensii. Marine Biotechnol (2012) 14(6):730–9. 10.1007/s10126-012-9438-3 22351046

[66] Huang J Li S Liu Y Liu C Xie L Zhang R. Hemocytes in the Extrapallial Space of Pinctada Fucata are Involved in Immunity and Biomineralization. Sci Rep (2018) 8(1). 10.1038/s41598-018-22961-y PMC5854705 29545643

[67] Kazama JJ Amizuka N Fukagawa M. Ectopic Calcification as Abnormal Biomineralization. Ther Apheresis Dialysis (2006) 10:S34–8. doi: 10.1111/j.1744-9987.2006.00438.x

[68] O’Grady S Morgan MP. Microcalcifications in Breast Cancer: From Pathophysiology to Diagnosis and Prognosis. Biochim Biophys Acta (BBA)-Rev Cancer (2018) 1869(2):310–20. doi: 10.1016/j.bbcan.2018.04.006 29684522

[69] Passos LSA Lupieri A Becker-Greene D Aikawa E. Innate and Adaptive Immunity in Cardiovascular Calcification. Atherosclerosis (2020) 306:59–67. doi: 10.1016/j.atherosclerosis.2020.02.016 32222287 PMC7483874

[70] Srivatsa SS Harrity PJ Maercklein PB Kleppe L Veinot J Edwards WD. Increased Cellular Expression of Matrix Proteins That Regulate Mineralization is Associated With Calcification of Native Human and Porcine Xenograft Bioprosthetic Heart Valves. J Clin Invest (1997) 99(5):996–1009. 9062358 10.1172/JCI119265 PMC507908

[71] Cao J Zu X Liu J. The Roles of Immune Cells in Atherosclerotic Calcification. Vascular (2021) p:17085381211032756. 10.1177/17085381211032756 34256610

[72] Vidavsky N Kunitake JA Estroff LA. Multiple Pathways for Pathological Calcification in the Human Body. Advanced Healthcare Materials (2021) 10(4):2001271. doi: 10.1002/adhm.202001271 PMC8724004 33274854

[73] Drake JL Mass T Haramaty L Zelzion E Bhattacharya D Falkowski PG. Proteomic Analysis of Skeletal Organic Matrix From the Stony Coral Stylophora Pistillata. Proc Natl Acad Sci (2013) 110(10):3788–93. 10.1073/pnas.1301419110 PMC3593878 23431140

[74] Goldberg M Kulkarni AB Young M Boskey A. Dentin: Structure, Composition and Mineralization: The Role of Dentin ECM in Dentin Formation and Mineralization. Front Biosci (Elite edition) (2011) 3:711. 10.2741/e281 PMC3360947 21196346

[75] Ohukainen P Näpäkangas J Ohtonen P Ruskoaho H Taskinen P Peltonen T. Expression and Localization of Granzymes and Perforin in Human Calcific Aortic Valve Disease. J Heart Valve Dis (2015) 24(5):612–20. 26897841

[76] Navas Madroñal M Castelblanco E Camacho M Consegal M Ramirez Morros A Sarrias M-R. Role of the Scavenger Receptor CD36 in Accelerated Diabetic Atherosclerosis. MDPI AG (2020). 10.3390/ijms21197360 PMC7583063 33028031

[77] Conus S. Cathepsins and Their Involvement in Immune Responses. Swiss Med Weekly (2010) 140:2930. 10.4414/smw.2010.13042 20648403

[78] Ellis A. Innate Host Defense Mechanisms of Fish Against Viruses and Bacteria. Dev Comp Immunol (2001) 25(8-9):827–39. 10.1016/s0145-305x(01)00038-6 11602198

[79] Ma J Zhang D Jiang J Cui S Pu H Jiang S. Molecular Characterization and Expression Analysis of Cathepsin L1 Cysteine Protease From Pearl Oyster Pinctada Fucata. Fish Shellfish Immunol (2010) 29(3):501–7. 10.1016/j.fsi.2010.05.006 20573562

[80] Shen J-D Cai Q-F Yan L-J Du C-H Liu G-M Su W-J. Cathepsin L is an Immune-Related Protein in Pacific Abalone (Haliotis Discus Hannai) – Purification and Characterization. Fish Shellfish Immunol (2015) 47(2):986–95. 10.1016/j.fsi.2015.11.004 26549175

[81] Wang X Hu S Gan L Wiens M Müller WE. Sponges (Porifera) as Living Metazoan Witnesses From the Neoproterozoic: Biomineralization and the Concept of Their Evolutionary Success Terra Nova (2010) 22:1–11.

[82] Iwata Y Mort JS Tateishi H Lee ER. Macrophage Cathepsin L, a Factor in the Erosion of Subchondral Bone in Rheumatoid Arthritis. Arthritis Rheumatism (1997) 40(3):499–509. 9082938 10.1002/art.1780400316

[83] Diment S Leech MS Stahl PD. Cathepsin D is Membrane-Associated in Macrophage Endosomes. J Biol Chem (1988) 263(14):6901–7. 3360812

[84] Wu H Du Q Dai Q Ge J Cheng X. Cysteine Protease Cathepsins in Atherosclerotic Cardiovascular Diseases. J Atheroscl Thromb (2017), RV17016. 10.5551/jat.RV17016 PMC5827079 28978867

[85] Andrault P-M Panwar P Mackenzie NCW Brömme D. Elastolytic Activity of Cysteine Cathepsins K, S, and V Promotes Vascular Calcification. Sci Rep (2019) 9(1). 10.1038/s41598-019-45918-1 PMC6609650 31273243

[86] Giansanti F Leboffe L Pitari G Ippoliti R Antonini G. Physiological Roles of Ovotransferrin. Biochim Biophys Acta (BBA) - Gen Subj (2012) 1820(3):218–25. 10.1016/j.bbagen.2011.08.004 21854833

[87] Valenti P Antonini G Von Hunolstein C Visca P Orsi N Antonini E. Studies of the Antimicrobial Activity of Ovotransferrin. Int J Tissue Reactions (1983) 5(1):97–105. 6345431

[88] Valenti P Visca P Antonini G Orsi N. Antifungal Activity of Ovotransferrin Towards Genus Candida. Mycopathologia (1985) 89(3):169–75. 10.1007/BF00447027 2985999

[89] Péterfi Z Donkó Á Orient A Sum A Prókai Á Molnár B. Peroxidasin Is Secreted and Incorporated Into the Extracellular Matrix of Myofibroblasts and Fibrotic Kidney. Am J Pathol (2009) 175(2):725–35. 10.2353/ajpath.2009.080693 PMC2716968 19590037

[90] Bhave G Cummings CF Vanacore RM Kumagai-Cresse C Ero-Tolliver IA Rafi M. Peroxidasin Forms Sulfilimine Chemical Bonds Using Hypohalous Acids in Tissue Genesis. Nat Chem Biol (2012) 8(9):784–90. doi: 10.1038/nchembio.1038 PMC4128002 22842973

[91] Ulfig A Leichert LI. The Effects of Neutrophil-Generated Hypochlorous Acid and Other Hypohalous Acids on Host and Pathogens. Cell Mol Life Sci (2021) 78(2):385–414. doi: 10.1007/s00018-020-03591-y 32661559 PMC7873122

[92] Parfitt DA Michael GJ Vermeulen EGM Prodromou NV Webb TR Gallo J-M. The Ataxia Protein Sacsin is a Functional Co-Chaperone That Protects Against Polyglutamine-Expanded Ataxin-1. Hum Mol Genet (2009) 18(9):1556–65. 10.1093/hmg/ddp067 PMC2667285 19208651

[93] Duncan EJ Cheetham ME Chapple JP van der Spuy J. The Role of HSP70 and its Co-Chaperones in Protein Misfolding, Aggregation and Disease. The Networking of Chaperones by Co-Chaperones (2015) p. 243–73. 10.1007/978-3-319-11731-7_12 25487025

[94] Workenhe ST Hori TS Rise ML Kibenge MJT Kibenge FSB. Infectious Salmon Anaemia Virus (ISAV) Isolates Induce Distinct Gene Expression Responses in the Atlantic Salmon (Salmo Salar) Macrophage/Dendritic-Like Cell Line TO, Assessed Using Genomic Techniques. Mol Immunol (2009) 46(15):2955–74. 10.1016/j.molimm.2009.06.015 19616850

[95] Lee JW Kim JE Goo IB Hwang J-A Im JH Choi H-S. Expression of Immune-Related Genes During Loach (Misgurnus Anguillicaudatus) Embryonic and Early Larval Development. Dev Reprod (2015) 19(4):181–7. 10.12717/DR.2015.19.4.181 PMC4786479 26973969

[96] Eslamloo K Xue X Booman M Smith NC Rise ML. Transcriptome Profiling of the Antiviral Immune Response in Atlantic Cod Macrophages. Dev Comp Immunol (2016) 63:187–205. 27255218 10.1016/j.dci.2016.05.021

[97] Hofman ER Boyanapalli M Lindner DJ Weihua X Hassel BA Jagus R. Thioredoxin Reductase Mediates Cell Death Effects of the Combination of Beta Interferon and Retinoic Acid. Mol Cell Biol (1998) 18(11):6493–504. 10.1128/mcb.18.11.6493 PMC109235 9774665

[98] Damdimopoulou PE Miranda-Vizuete A Arnér ESJ Gustafsson J-Å Damdimopoulos AE. The Human Thioredoxin Reductase-1 Splice Variant TXNRD1_v3 is an Atypical Inducer of Cytoplasmic Filaments and Cell Membrane Filopodia. Biochim Biophys Acta (BBA) - Mol Cell Res (2009) 1793(10):1588–96. 10.1016/j.bbamcr.2009.07.007 19654027

[99] Vandermeulen J Watabe N. Studies on Reef Corals. I. Skeleton Formation by Newly Settled Planula Larva of Pocillopora Damicornis. Marine Biol (1973) 23(1):47–57.

[100] Mor Khalifa G Levy S Mass T. The Calcifying Interface in a Stony Coral Primary Polyp: An Interplay Between Seawater and an Extracellular Calcifying Space. J Struct Biol (2021), 107803. 34695544 10.1016/j.jsb.2021.107803 PMC7611985

[101] Prajapati S Tao J Ruan Q De Yoreo JJ Moradian-Oldak J. Matrix Metalloproteinase-20 Mediates Dental Enamel Biomineralization by Preventing Protein Occlusion Inside Apatite Crystals. Biomaterials (2016) 75:260–70. 10.1016/j.biomaterials.2015.10.031 PMC4654413 26513418

[102] Morgulis M Winter MR Shternhell L Gildor T Ben-Tabou De-Leon S. VEGF Signaling Activates the Matrix Metalloproteinases, MmpL7 and MmpL5 at the Sites of Active Skeletal Growth and MmpL7 Regulates Skeletal Elongation. Dev Biol (2021) 473:80–9. 10.1016/j.ydbio.2021.01.013 33577829

[103] Kato K Chu P Takahashi S Hamada H Kipps TJ. Metalloprotease Inhibitors Block Release of Soluble CD27 and Enhance the Immune Stimulatory Activity of Chronic Lymphocytic Leukemia Cells. Exp Hematol (2007) 35(3):434–42. 10.1016/j.exphem.2006.10.018 17309824

[104] Menaldo DL. Immune Cells and Mediators Involved in the Inflammatory Responses Induced by a P-I Metalloprotease and a Phospholipase A2 from Bothrops Atrox Venom. Mol Immunol (2017) 85:238–47. 10.1016/j.molimm.2017.03.008 28327442

[105] Patel S. A Critical Review on Serine Protease: Key Immune Manipulator and Pathology Mediator. Allergologia Immunopathol (2017) 45(6):579–91. 10.1016/j.aller.2016.10.011 PMC7126602 28236540

[106] Soria-Valles C Gutiérrez-Fernández A Osorio FG Carrero D Ferrando AA Colado E. MMP-25 Metalloprotease Regulates Innate Immune Response Through NF-κb Signaling. J Immunol (2016) 197(1):296–302. 27259858 10.4049/jimmunol.1600094

