# Supplementary material for: The Skeleton and Biomineralization Mechanism as Part of the Innate Immune System of Stony Corals
Source: Front Immunol. 2022 Feb 25;13:850338. doi: 10.3389/fimmu.2022.850338 (PMC8913943; doi:10.3389/fimmu.2022.850338)
Supplement: Supplementary file 1 [file Table_1.pdf]

Supporting Information:

The Skeleton and Biomineralization Mechanism as part of the Innate Immune System of Stony Corals

Shani Levy[1,2*], Tali Mass[1,2*]

[1]Department of Marine Biology, Leon H. Charney School of Marine Sciences, University of Haifa, Haifa, Israel

[2]Morris Kahn Marine Research Station, The Leon H. Charney School of Marine Sciences, University of Haifa, Sdot Yam, Israel

*** Correspondence:**
Corresponding Author
Shani Levy, levyshani78@gmail.com
Tali Mass, tmass@univ.haifa.ac.il

Table S1: Candidate genes with potential dual function in the coral's innate immune response and in biomineralization.

Gene name	Organism	Function in the immune response	Function in biomineralization	Homolog in corals	Expression in corals	References
**TYR** (Tyrosinase)	Arthropods	Melanin production. Antimicrobial activity.	Melanin production. Strengthens exoskeletons to improve their ability to act as physical barriers against parasites penetration.	XP_022801515.1<br>XP_022801546.1<br>XP_022797084.1	Immune cells & Calicoblasts<br>Calicoblasts<br>Calicoblasts	(St. Leger, Cooper, and Charnley 1988)<br>(Mackintosh 2001)
**Meprin A**	Humans & mice	A metallopeptidase that processes proinflammatory cytokines and promotes leukocyte infiltration.	Involved in extracellular degradation and matrix remodeling.	XP_022785469.1	Calicoblasts	(Kaushal et al. 2013)
**Mucin-like**	-	Comprises SHK domains, therefore it might have a toxic activity.	-	XP_022806382.1	Calicoblast	-
**SPP1** (Osteopontin)	Mammals	Acts as a cytokine. Enhances adaptive immune cells production and proliferation. Stimulates both pro- and anti-inflammatory processes.	A secreted protein that binds hydroxyapatite with high affinity. Forms an integral part of the mineralized matrix of bones.	No homolog	-	(Caputo and Bellone 2018)<br>(Foster et al. 2018)

**Perforin 1**	Mammals	Pore-forming protein. Promotes cytolysis and apoptosis of target cells by facilitating the uptake of cytotoxic granzymes.	Increased expression in calcified regions of aortic valve.	XP_022807111.1	Immune cells	(Hansson and Hermansson 2011; Gupta et al. 2020) (Ohukainen et al. 2015)
**CD36** (CD36 Molecule)	Humans	Promotes inflammation.	Functions as a cell adhesion molecule. Binds to collagen, thrombospondin, fatty acids, and oxidized LDL. Enhances vascular calcification.	XP_022800213.1	Immune cells	(Navas Madroñal et al. 2020)
**DOCK1** (Dedicator Of Cytokinesis 1)	Humans	Involved in cytoskeletal rearrangements. Required for phagocytosis.	Actin remodeling. Enhances bone resorption and inhibits bone formation.	XP_022795721.1	Immune cells	(Kukimoto-Niino et al. 2021) (Laurin and Côté 2014)
**DSPP** (Dentin Sialophosphoprotein)	Mammals	Unknown	Secreted by odontoblasts. Binds calcium and facilitates initial mineralization of dentin matrix collagen. Regulates the size and shape of the crystals.	XP_022793445.1<br>XP_022802034.1<br>XP_022806140.1<br>XP_022807057.1	Gland cells<br>Immune cells<br>Cnidocytes<br>Cnidocytes & Gland cells	(Goldberg et al. 2011)
**Cathepsin-F**	Mice	Involved in the degradation of the invariant chain of MHC class II complexes in antigen-presenting cells.	Unknown	XP_022799019.1	Immune cells	(Shi et al. 2000)
**Cathepsin-Z**	Mice	Restricted predominantly to the immune cells. Responsible for the adhesion and migration of immune cells. Leads to the production of cytokines that promote inflammation (as IL-1β).	Unknown	XP_022790465.1	Immune cells & calicoblasts	(Batista et al. 2021) (Campden et al. 2022)
**Cathepsin-B**	Humans	Processing of antigens in the immune response.	Participates in the degradation of cartilage matrix prior to the synthesis of bone matrix proteins. Involved in biomineralization inhibition and in bone turnover.	XP_022795065.1<br>XP_022802777.1	Immune cells & calicoblasts<br>Immune cells	(Friemert et al. 1991)
**Cathepsin-L**	Humans, Fish, and Mollusks	High expression following infection.	Involved in bone and cartilage resorption. Involved in vascular calcification.	XP_022798828.1	Immune cells & calicoblasts	(Shen et al. 2015) (Ellis 2001) (Ma et al. 2010) (Iwata, Mort et al. 1997)

This is a provisional file, not the final typeset article

**Cathepsin-V**	Humans & mice	Might be involved in T-cell selection.	Promotes vascular calcification.	XP_022798839.1	Calicoblasts	(Andrault et al. 2019)
**Cathepsin-D**	Humans	Found in macrophage endosomes. An activator of cathepsin B.	Aspartic protease. Involved in cardiovascular calcification.	XP_022781957.1	Immune cells	(Rowe et al. 2006)
**CDCP1**<br>(CUB and peptidase domain-<br>containing protein 1)	-	Unknown	Unknown	B8V7S0	*A. millepora* skeletal proteome	(Ramos-Silva et al. 2013)
**CDCP2**<br>(CUB and peptidase domain-<br>containing protein 2)	-	Unknown	Unknown	B8VIV4	*A. millepora* skeletal proteome	(Ramos-Silva et al. 2013)
**Serine Protease 15**	-	Unknown	Unknown	XP_022788730.1	*S. pistillata* skeletal proteome & calicoblasts	(Peled et al. 2020)
**Serine protease 23**	-	Unknown	Unknown	XP_022807249.1	*S. pistillata* skeletal proteome & calicoblasts	(Peled et al. 2020)
**MPLs**<br>(Metallopeptidases)	Mammals & marine invertebrates	Processing enzymes of proinflammatory cytokines.	Processing and activating factors involved in biomineralization.<br>involved in degradation of the extracellular matrix (ECM).	XP_022784314.1<br>XP_022784340.1<br>XP_022803140.1<br>XP_022802289.1<br>XP_022797863.1<br>XP_022803576.1<br>XP_022788159.1<br>XP_022798355.1<br>XP_022783952.1<br>XP_022788159.1	Calicoblasts<br>Calicoblasts<br>Calicoblasts<br>Calicoblasts<br>Calicoblasts<br>Calicoblasts<br>Calicoblasts<br>Calicoblasts<br>Calicoblasts<br>Calicoblasts	(Passos et al. 2020)<br>(Menaldo et al. 2017)<br>(Morgulis et al. 2021)
**PXDN**<br>(Peroxidasin)	Vertebrates & Invertebrates	Catalyzes generation of hypohalous acids that kill bacteria.	The protein is secreted into the ECM, where it becomes organized into a fibril-like network and colocalizes with fibronectin to form the extracellular matrix	XP_022794431.1	Calicoblasts	(Péterfi et al. 2009)<br>(Ulfig and Leichert 2021)<br>(Bhave et al. 2012)
**Ovotransferrin**	Birds	Antimicrobial and antifungal activity.	Involved in eggshell formation and calcite nucleation.	XP_022780954.1	Calicoblasts	(Legros et al. 2021)
**Sacsin**	Fish	Involved in the innate antiviral immune response.	Unknown	XP_022808646.1<br>/PFX13778.1	Skeleton proteome	(Workenhe et al. 2009)<br>(Lee et al. 2015)

**TXNRD1**<br>(Thioredoxin reductase 1)		Mediates cell death.	Unknown.<br>Induces actin and tubulin polymerization.	XP_022804785.1	Skeleton proteome	(Anestål et al. 2008)<br>(Damdimopoulou et al. 2009)
**RUNX2**<br>(Runt-related transcription factor 2)	Humans and mice	Important for the long-term memory of T-cells.	Essential for osteoblast differentiation and chondrocyte maturation.	XP_022805311.1	Calicoblasts & neurons	(Olesin et al. 2018)
**TLRs**<br>(Toll-like receptors)	Humans and mice	Pattern Recognition Receptors (PRRs). Detection of potential harmful pathogens and activation of the innate immune response<br>And inflammatory response.	Promotes vascular calcification	XP_022794422.1	Neurons & epidermal cells	(Passos et al. 2020)
**IL1R1**<br>(Interleukin-1 Receptor type 1)	Humans and mice	Activates NF-κB pathway to promote immune & inflammatory response.	Unknown	XP_022778509.1	Immune cells & calicoblasts	(Subramaniam, Stansberg, and Cunningham 2004)
**TNF-α**<br>(Tumor Necrosis Factor alpha)	Mammals	Proinflammatory cytokine. Induces necrosis and apoptosis.	Regulator of bone formation. Induces Vascular smooth muscle cells (VSMCs) differentiation into an osteochondrogenic phenotype, releasing calcifying EVs.	XP_022801114.1<br>XP_022790994.1	Calicoblasts<br>Neurons	(Tintut et al. 2000)<br>(Passos et al. 2020)
**NF-κB**<br>(Nuclear Factor Kappa B)	Humans	Transcription factor. Induces inflammatory gene expression.	Activation of NF-κB by TNF promote calcification in human aortic smooth muscle cells.	XP_022798721.1<br>XP_022801431.1	Immune cells & epidermal cells<br>Neurons & epidermal cells	(Passos et al. 2020)
**NFAT**<br>(Nuclear factor of activated T-cells)	Mice	Transcription factor. Key regulator of T-cells activation, differentiation, and development. Induces gene expression during immune response.	Regulates genes important for osteoclast differentiation and function.	XP_022795880.1	Immune cells	(Macian 2005)<br>(Takayanagi 2007)
**IL-6**<br>(Interleukin 6)	Humans	Proinflammatory cytokine. Plays a major role in the proliferation and differentiation of immune cells. A potent inducer of the acute phase response.	Promotes osteoclast activity. promotes<br>osteogenic differentiation and mineralization of VSMCs.	No homolog	-	(Passos et al. 2020)

This is a provisional file, not the final typeset article

References

Andrault, Pierre-Marie, Preety Panwar, Neil C. W. Mackenzie, and Dieter Brömme. 2019. 'Elastolytic activity of cysteine cathepsins K, S, and V promotes vascular calcification', *Scientific Reports*, 9.

Anestål, Karin, Stefanie Prast-Nielsen, Narimantas Cenas, and Elias SJ Arnér. 2008. 'Cell death by SecTRAPs: thioredoxin reductase as a prooxidant killer of cells', *PLoS ONE*, 3: e1846.

Batista, A. A. S., B. M. Franco, M. M. Perez, E. G. Pereira, T. Rodrigues, M. L. Wroclawski, F. L. A. Fonseca, and E. R. Suarez. 2021. 'Decreased levels of cathepsin Z mRNA expressed by immune blood cells: diagnostic and prognostic implications in prostate cancer', *Brazilian Journal of Medical and Biological Research*, 54.

Bhave, Gautam, Christopher F. Cummings, Roberto M. Vanacore, Chino Kumagai-Cresse, Isi A. Ero-Tolliver, Mohamed Rafi, Jeong-Suk Kang, Vadim Pedchenko, Liselotte I. Fessler, John H. Fessler, and Billy G. Hudson. 2012. 'Peroxidasin forms sulfilimine chemical bonds using hypohalous acids in tissue genesis', *Nature Chemical Biology*, 8: 784-90.

Campden, Rhiannon I., Amy L. Warren, Catherine J. Greene, Jose A. Chiriboga, Corey R. Arnold, Devin Aggarwal, Neil McKenna, Christina F. Sandall, Justin A. Macdonald, and Robin M. Yates. 2022. 'Extracellular cathepsin Z signals through the α5 integrin and augments NLRP3 inflammasome activation', *Journal of Biological Chemistry*, 298: 101459.

Caputo, Sara, and Matteo Bellone. 2018. 'Osteopontin and the immune system: another brick in the wall', *Cellular & molecular immunology*, 15: 405-07.

Damdimopoulou, Pauliina E., Antonio Miranda-Vizuete, Elias S. J. Arnér, Jan-Åke Gustafsson, and Anastasios E. Damdimopoulos. 2009. 'The human thioredoxin reductase-1 splice variant TXNRD1_v3 is an atypical inducer of cytoplasmic filaments and cell membrane filopodia', *Biochimica et Biophysica Acta (BBA) - Molecular Cell Research*, 1793: 1588-96.

Ellis, AE. 2001. 'Innate host defense mechanisms of fish against viruses and bacteria', *Developmental & Comparative Immunology*, 25: 827-39.

Foster, BL, M Ao, CR Salmon, MB Chavez, TN Kolli, AB Tran, EY Chu, KR Kantovitz, M Yadav, and S Narisawa. 2018. 'Osteopontin regulates dentin and alveolar bone development and mineralization', *Bone*, 107: 196-207.

Friemert, Constanze, Ellen I. Closs, Michael Silbermann, Volker Erfle, and P. Günter Strauss. 1991. 'Isolation of a cathepsin B-encoding cDNA from murine osteogenic cells', *Gene*, 103: 259-61.

Goldberg, Michel, Askok B Kulkarni, Marian Young, and Adele Boskey. 2011. 'Dentin: Structure, Composition and Mineralization: The role of dentin ECM in dentin formation and mineralization', *Frontiers in bioscience (Elite edition)*, 3: 711.

Gupta, Shashi Kumar, Sunaina Kumari, Sandhya Singh, Manoj Kumar Barthwal, Sushil Kumar Singh, and Thomas Thum. 2020. 'Non-coding RNAs: Regulators of valvular calcification', *Journal of Molecular and Cellular Cardiology*, 142: 14-23.

Hansson, Göran K., and Andreas Hermansson. 2011. 'The immune system in atherosclerosis', *Nature Immunology*, 12: 204-12.

Kaushal, Gur P, Randy S Haun, Christian Herzog, and Sudhir V Shah. 2013. 'Meprin A metalloproteinase and its role in acute kidney injury', *American Journal of Physiology-Renal Physiology*, 304: F1150-F58.

Kukimoto-Niino, Mutsuko, Kentaro Ihara, Kazutaka Murayama, and Mikako Shirouzu. 2021. 'Structural insights into the small GTPase specificity of the DOCK guanine nucleotide exchange factors', *Current opinion in structural biology*, 71: 249-58.

Laurin, Mélanie, and Jean-François Côté. 2014. 'Insights into the biological functions of Dock family guanine nucleotide exchange factors', *Genes & development*, 28: 533-47.

Lee, Jang Wook, Jung Eun Kim, In Bon Goo, Ju-Ae Hwang, Jea Hyun Im, Hye-Sung Choi, and Jeong-Ho Lee. 2015. 'Expression of Immune-Related Genes during Loach (Misgurnus anguillicaudatus) Embryonic and Early Larval Development', *Development & Reproduction*, 19: 181-87.

Legros, Julie, Sophie Jan, Sylvie Bonnassie, Michel Gautier, Thomas Croguennec, Stéphane Pezennec, Marie-Françoise Cochet, Françoise Nau, Simon C. Andrews, and Florence Baron. 2021. 'The Role of Ovotransferrin in Egg-White Antimicrobial Activity: A Review', *Foods*, 10: 823.

Ma, Jianjun, Dianchang Zhang, Jingjing Jiang, Shuge Cui, Hanlin Pu, and Shigui Jiang. 2010. 'Molecular characterization and expression analysis of cathepsin L1 cysteine protease from pearl oyster Pinctada fucata', *Fish & Shellfish Immunology*, 29: 501-07.

Macian, Fernando. 2005. 'NFAT proteins: key regulators of T-cell development and function', *Nature Reviews Immunology*, 5: 472-84.

Mackintosh, James A. 2001. 'The antimicrobial properties of melanocytes, melanosomes and melanin and the evolution of black skin', *Journal of theoretical biology*, 211: 101-13.

Menaldo, Danilo L., Carolina P. Bernardes, Karina F. Zoccal, Anna L. Jacob-Ferreira, Tássia R. Costa, Maria P. F. M. Del Lama, Rose M. Z. G. Naal, Fabiani G. Frantz, Lúcia H. Faccioli, and Suely V. Sampaio. 2017. 'Immune cells and mediators involved in the inflammatory responses induced by a P-I metalloprotease and a phospholipase A2 from Bothrops atrox venom', *Molecular Immunology*, 85: 238-47.

Morgulis, Miri, Mark R. Winter, Ligal Shternhell, Tsvia Gildor, and Smadar Ben-Tabou De-Leon. 2021. 'VEGF signaling activates the matrix metalloproteinases, MmpL7 and MmpL5 at the sites of active skeletal growth and MmpL7 regulates skeletal elongation', *Developmental Biology*, 473: 80-89.

Navas Madroñal, Miquel, Esmeralda Castelblanco, Mercedes Camacho, Marta Consegal, Anna Ramirez Morros, Maria-Rosa Sarrias, Paulina Perez, Nuria Alonso, María Galán, and Dídac Mauricio. 2020. "Role of the Scavenger Receptor CD36 in Accelerated Diabetic Atherosclerosis." In.: MDPI AG.

Ohukainen, Pauli, Juha Näpäkangas, Pasi Ohtonen, Heikki Ruskoaho, Panu Taskinen, Tuomas Peltonen, and Jaana Rysä. 2015. 'Expression and Localization of Granzymes and Perforin in Human Calcific Aortic Valve Disease', *The Journal of heart valve disease*, 24: 612-20.

Olesin, Elizabeth, Ribhu Nayar, Priya Saikumar-Lakshmi, and Leslie J. Berg. 2018. 'The Transcription Factor Runx2 Is Required for Long-Term Persistence of Antiviral CD8+ Memory T Cells', *ImmunoHorizons*, 2: 251-61.

Passos, Livia S. A., Adrien Lupieri, Dakota Becker-Greene, and Elena Aikawa. 2020. 'Innate and adaptive immunity in cardiovascular calcification', *Atherosclerosis*, 306: 59-67.

This is a provisional file, not the final typeset article

Peled, Yanai, Jeana L. Drake, Assaf Malik, Ricardo Almuly, Maya Lalzar, David Morgenstern, and Tali Mass. 2020. 'Optimization of skeletal protein preparation for LC–MS/MS sequencing yields additional coral skeletal proteins in Stylophora pistillata', *BMC Materials*, 2.

Péterfi, Zalán, Ágnes Donkó, Anna Orient, Adrienn Sum, Ágnes Prókai, Beáta Molnár, Zoltán Veréb, Éva Rajnavölgyi, Krisztina J. Kovács, Veronika Müller, Attila J. Szabó, and Miklós Geiszt. 2009. 'Peroxidasin Is Secreted and Incorporated into the Extracellular Matrix of Myofibroblasts and Fibrotic Kidney', *The American Journal of Pathology*, 175: 725-35.

Ramos-Silva, Paula, Jaap Kaandorp, Lotte Huisman, Benjamin Marie, Isabelle Zanella-Cléon, Nathalie Guichard, David J. Miller, and Frédéric Marin. 2013. 'The Skeletal Proteome of the Coral Acropora millepora: The Evolution of Calcification by Co-Option and Domain Shuffling', *Molecular Biology and Evolution*, 30: 2099-112.

Rowe, Peter S. N., Naoko Matsumoto, Oak D. Jo, Remi N. J. Shih, Jeannine Oconnor, Martine P. Roudier, Steve Bain, Shiguang Liu, Jody Harrison, and Norimoto Yanagawa. 2006. 'Correction of the mineralization defect in hyp mice treated with protease inhibitors CA074 and pepstatin', *Bone*, 39: 773-86.

Shen, Jian-Dong, Qiu-Feng Cai, Long-Jie Yan, Cui-Hong Du, Guang-Ming Liu, Wen-Jin Su, Caihuan Ke, and Min-Jie Cao. 2015. 'Cathepsin L is an immune-related protein in Pacific abalone (Haliotis discus hannai) – Purification and characterization', *Fish & Shellfish Immunology*, 47: 986-95.

Shi, Guo-Ping, Rebecca A. R. Bryant, Richard Riese, Steven Verhelst, Christoph Driessen, Zhenqiang Li, Dieter Bromme, Hidde L. Ploegh, and Harold A. Chapman. 2000. 'Role for Cathepsin F in Invariant Chain Processing and Major Histocompatibility Complex Class II Peptide Loading by Macrophages', *Journal of Experimental Medicine*, 191: 1177-86.

St. Leger, R. J., R. M. Cooper, and A. K. Charnley. 1988. 'The effect of melanization of Manduca sexta cuticle on growth and infection by Metarhizium anisopliae', *Journal of Invertebrate Pathology*, 52: 459-70.

Subramaniam, Sumathi, Christine Stansberg, and Charles Cunningham. 2004. 'The interleukin 1 receptor family', *Developmental & Comparative Immunology*, 28: 415-28.

Takayanagi, Hiroshi. 2007. 'The role of NFAT in osteoclast formation', *Annals of the New York Academy of Sciences*, 1116: 227-37.

Tintut, Yin, Jignesh Patel, Farhad Parhami, and Linda L. Demer. 2000. 'Tumor Necrosis Factor-α Promotes In Vitro Calcification of Vascular Cells via the cAMP Pathway', *Circulation*, 102: 2636-42.

Ulfig, Agnes, and Lars I. Leichert. 2021. 'The effects of neutrophil-generated hypochlorous acid and other hypohalous acids on host and pathogens', *Cellular and Molecular Life Sciences*, 78: 385-414.

Workenhe, Samuel T., Tiago S. Hori, Matthew L. Rise, Molly J. T. Kibenge, and Frederick S. B. Kibenge. 2009. 'Infectious salmon anaemia virus (ISAV) isolates induce distinct gene expression responses in the Atlantic salmon (Salmo salar) macrophage/dendritic-like cell line TO, assessed using genomic techniques', *Molecular Immunology*, 46: 2955-74.

Iwata, Y., J. S. Mort, H. Tateishi and E. R. Lee (1997). "Macrophage cathepsin L, a factor in the erosion of subchondral bone in rheumatoid arthritis." Arthritis & Rheumatism **40**(3): 499-509.

This is a provisional file, not the final typeset article
